# Enhancing emergency department experiences for older adults: a rapid review (2020–2025)

**DOI:** 10.1186/s12245-026-01219-9

**Published:** 2026-04-14

**Authors:** Jae-Yung Kwon, Mariko Sakamoto, Kayoung Lee, Alyssa DeMedeiros, Simrit Sandhu, Kira Gossack-Keenan

**Affiliations:** 1 School of Nursing, University of Victoria, Victoria, BC Canada; 2 Institute on Aging and Lifelong Health, Victoria, BC Canada; 3 School of Nursing, University of British Columbia, Vancouver, BC Canada; 4 Department of Emergency Medicine, University of British Columbia, Vancouver, BC Canada

**Keywords:** Geriatric emergency care, Older adults, Patient experience, Person-centred interventions, Quality improvement

## Abstract

**Objectives:**

Older adults (65+) account for nearly a quarter of all emergency department (ED) visits, yet consistently report challenges in care experiences, including communication gaps, long wait times, and a lack of age-appropriate services. This systematic rapid review synthesizes recent evidence on ED interventions that improved older adults’ care experiences.

**Methods:**

We conducted the review guided by the Joanna Briggs Institute convergent integrated approach. MEDLINE, CINAHL, Web of Science, and AgeLine were searched (January 2020-July 2025). Eligible studies examined ED-based interventions for older adults ≥ 65 years and reported patient experience outcomes. Three reviewers independently screened, extracted, and appraised studies using the Mixed Methods Appraisal Tool. Findings were synthesized thematically across intervention domains.

**Results:**

Ten studies met the inclusion criteria (i.e., 5 quantitative, 2 mixed-methods, and 3 qualitative). Interventions included multidisciplinary geriatric teams, frailty-specific pathways, redesigned discharge tools, comfort carts, assistive devices, advance care planning, and elder mistreatment screening. Interventions improved satisfaction, dignity, independence, and perceptions of care quality across four countries (Australia, Ireland, the Netherlands, and the United States of America). Frailty-focused programs and trauma-informed screening were broadly acceptable when framed respectfully. Structured communication redesigns and environmental modifications enhanced patient trust and comfort. Common limitations included small samples and single-site designs.

**Conclusions:**

Embedding geriatric expertise, structured communication, environmental supports, and trauma-informed practices improves older adults’ perceptions of ED experiences. Future research should prioritize multi-site evaluations, cost-effectiveness, and integration of patient-reported experience measures into routine monitoring.

## Introduction

Globally, adults aged 65 years and older comprise a rapidly growing proportion of the population and account for a disproportionately high share of emergency department (ED) visits, often exceeding a quarter of all presentations in high-income countries [1]. As the population continues to age, EDs will increasingly serve as critical entry points to acute care for older adults, highlighting the need to tailor ED practices and infrastructure to this population. Older adults and clinicians alike have identified persistent challenges in ED care, including prolonged wait times, inadequate communication, poor transitions between care settings, and insufficient age-appropriate services [2, 3]. These issues not only compromise clinical outcomes but also affect the dignity, autonomy, and overall care experience of older adults. The COVID-19 pandemic further strained ED operations while simultaneously accelerating innovation. Emerging interventions include age-friendly EDs, targeted communication supports, environmental modifications, virtual care enhancements, and restructured triage and waiting room processes. While previous reviews have examined interventions to improve ED care [1, 4], most predate the pandemic, and few specifically foreground the experiences of older adults. Given the pace of change, there is a pressing need to synthesize recent evidence through the lens of patient experience, a core indicator of person-centred quality care [5]. Accordingly, this systematic rapid review focuses on emergency department interventions implemented to improve older adult experiences from 2020 to 2025.

## Methods

### Design

We conducted a systematic rapid review using the Joanna Briggs Institute convergent integrated approach to synthesize findings [6]. A rapid review methodology was selected to provide timely, evidence-informed insights to support an ongoing regional initiative to improve ED care for older adults. The findings were needed within the project timeline to inform regional workshops with patient partners, clinicians, and healthcare administrators. Compared with a full systematic review, rapid reviews use streamlined yet transparent methods to accelerate evidence synthesis while maintaining methodological rigour. The review protocol was registered with PROSPERO (CRD420251019511).

### Database searches

We searched MEDLINE, CINAHL, Web of Science, and AgeLine for studies published between January 1, 2020, and July 2, 2025. MEDLINE and Web of Science were selected for broad biomedical and health services coverage, CINAHL for nursing and allied health literature, and AgeLine for gerontology-specific evidence. Search strategies were developed and reviewed in consultation with a university librarian (see Appendix for the full search strings). Inclusion criteria were (1) ED-based interventions involving older adults (≥ 65 years), (2) quantitative, mixed methods, or qualitative designs, and (3) studies reporting patient experience outcomes, defined as patient-reported perceptions of care during the ED encounter, such as satisfaction with care, communication with clinicians, perceived quality of care, or involvement in decision-making. Outcomes had to be reported directly by patients through surveys or interviews. Exclusion criteria included (1) non-ED settings, (2) studies reporting only clinical or utilization outcomes without patient experience outcomes, (3) non-English publications, and (4) non-peer-reviewed studies, review articles, editorials, and grey literature.

### Screening, data extraction, and quality assessment

All titles, abstracts, and full texts were screened independently by three reviewers (JY, MS, KL) using Covidence, a web-based platform for conducting reviews [7]. Two reviewers screened each abstract, with discrepancies resolved by a third reviewer. Inter-rater reliability was assessed using Cohen’s kappa [8]. For title and abstract screening, agreement between reviewer pairs ranged from κ = 0.33 to 0.57, with percent agreement ranging from 96.0% to 99.1%. For full-text screening, agreement between two reviewers (JY and KL) was very high (κ = 0.86; 95.5% agreement).

**Fig. 1:**
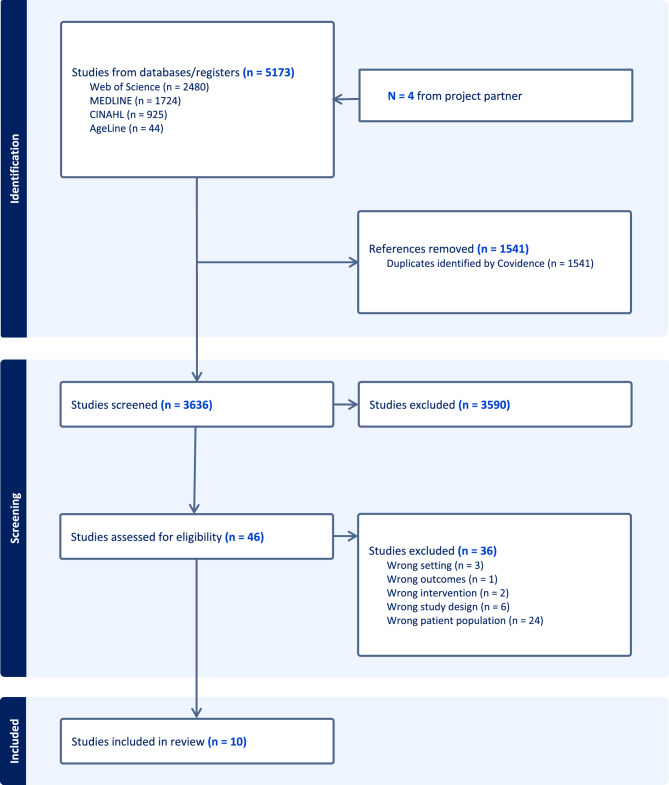
PRISMA flowchart of the selected studies

The search yielded 5,173 records, plus 4 records from health authority project partners; after removing 1,541 duplicates, 3,636 records remained for title and abstract screening. Of these, only 46 records met the inclusion criteria at the abstract stage and were assessed in full text. At the full-text review, 36 articles were excluded for the following reasons: wrong setting (*n* = 3, interventions not in the ED), wrong outcomes (*n* = 1, did not include patient experience), wrong intervention (*n* = 2, not delivered in the ED), wrong study design (*n* = 6, commentaries or reviews), and wrong patient population (*n* = 24, participants younger than 65 years). Ultimately, 10 studies met the inclusion criteria. A PRISMA flow diagram summarizes the selection process (see Fig. 1). Data from the 10 studies were extracted into a structured form, capturing study design, setting, population characteristics, intervention details, and patient experience outcomes. These studies were appraised using the Mixed Methods Appraisal Tool (MMAT) to assess the methodological quality of quantitative, mixed methods, and qualitative studies [9, 10]. Inter-rater reliability for the MMAT across all ratings was 87.1%, with κ = 0.64, indicating substantial agreement between reviewers. Discrepancies were resolved through discussion among the reviewers.

### Data synthesis and analysis

Findings were synthesized thematically by intervention type. Quantitative results were summarized descriptively and, where applicable, grouped by intervention type. Qualitative and mixed methods data were analysed inductively to identify recurring patterns in patient perspectives. Finally, following a convergent integrated approach, both quantitative and qualitative evidence were integrated under common themes based on intervention type to construct a narrative synthesis.

## Results

### Study characteristics

Ten studies (2020–2025) spanned diverse geographic settings in 4 countries (1 Australia, 1 Ireland, 2 Netherlands, 6 United States). Designs included 5 quantitative (1 randomized controlled trial, 3 non-randomized, and 1 descriptive), 2 mixed-methods, 3 qualitative. Sample size ranged from 4 to 821 participants, aged 65 to 94. Interventions ranged from creating specialized care teams and using assistive technologies to redesigning discharge processes and screening protocols. Primary outcomes assessed included patient experience, satisfaction, hospital admissions, ED revisits, and functional or psychosocial wellbeing (see Table 1).

**Table 1:** Study characteristics

Author/ Year	Country	Study Design/ Sample Size (*N*)	Demographics	Intervention	Outcomes on Patient Experience
Blomaard et al. (2021) [11]	Netherlands	Qualitative; *N* = 13 ED patients ≥ 70 years screened with Acutely Presenting Older Patient screener	Median age 82 years (range 71–94); 54% female; 46% high-risk screening result (functional decline or cognitive impairment)	Routine geriatric/frailty screening at ED triage	Screening seen as normal and holistic; improved communication and comfort; no perceived age discrimination
Burnett et al. (2024) [12]	United States	Qualitative; *N* = 19 older adults (≥ 65) with Adult Protective Services validated elder mistreatment cases	Mean age 74 years (range 65–91); 63% female; mistreatment types: physical (67%), emotional (17%), financial (11%), sexual (5%)	Elder mistreatment screening/response practices in ED (EM-SART)	Screening acceptable if private and compassionate; survivors valued rapport, transparency, and shared decision-making; concerns about stigma, safety, and autonomy
Cassarino et al. (2021) [13]	Ireland	Quantitative randomized controlled trial; *N* = 353 ED patients ≥ 65 with lower urgency presentations	Mean age 79.6 ± 7 years; 59% female; 78.7% at risk of adverse outcomes (ISAR ≥ 2); 53% had falls in previous 3 months; 32% hospitalized in prior 6 months	Health and social care professional (HSCP) team (social worker, occupational therapist, physiotherapist)	Reduced ED length of stay (6.4 vs. 12.1 h); reduced admissions (19.3% vs. 55.9%); higher satisfaction and better functional outcomes at 30 days and 6 months
Carayon et al. (2025) [14]	United States	Mixed methods; *N* = 15 older adults aged ≥ 65 years discharged from ED	Age ≥ 65 years; 47% female; all participants presented with fall-related ED visits and were discharged home	Patient-centered discharge redesign (After Visit Summaries (AVS), Electronic health record template, nurse teach-back)	Improved comprehension/satisfaction; usability valued by staff; gaps remained in information completeness
Fareed et al. (2021) [15]	United States	Qualitative; *N* = 9 ED patients aged ≥ 65 years and *N* = 15 ED staff	Mean patient age 78 ± 10 years; 45% female; older adults receiving geriatric ED assistive devices	Assistive devices (walkers, hearing amplifiers, cognitive engagement tools – puzzles and fidget toys)	Improved independence, dignity, and engagement; safer discharge; staff reported enhanced patient empowerment
Harper et al. (2023) [16]	Australia	Quantitative non-randomized; *N* = 192 older women ≥ 70 years discharged from ED	Mean age 83.6 ± 6.8 years; 100% female; mild frailty (Clinical Frailty Scale 3–5); 51.6% lived alone; 39.6% reported falls in the past year	Frailty Intervention Team (FIT) – frailty screening, allied health interventions	90% patient satisfaction; 83% accepted frailty strategies; barriers in communication and implementation post-discharge
Lichen et al. (2021) [17]	United States	Quantitative descriptive; *N* = 300 older ED patients aged ≥ 65 years, and *N* = 100 providers	Patients aged ≥ 65 years receiving ED care; older adults with sensory and communication challenges common in ED settings	Comfort cart with non-pharmacologic aids (e.g., hearing amplifiers, glasses, blankets)	98% patients and 95% providers reported improved comfort, satisfaction, communication, and independence
Liberman et al. (2020) [18]	United States	Quantitative non-randomized; *N* = 283 older adults aged ≥ 65 years discharged home, matched with historical usual-care cohort (*n* = 283)	Age ≥ 65 years; 78% female; community-dwelling older adults with complex comorbidities; 37% history of falls; 19% dementia; 14% stroke	Geriatric and palliative (GAP-ED) specialist placed in the ED	High patient/family satisfaction; reduced hospitalizations from 30-day revisits; no change in revisit rates
Ouchi et al. (2024) [19]	United States	Quantitative non-randomized; *N* = 26 patient-caregiver dyads aged ≥ 50 years, cognitive impairment)	Mean age 79 ± 8.5 years; 65% female; 69% moderate dementia; patients with cognitive impairment participating with caregivers	Advance care planning (ACP) motivational interview (ED-GOAL)	77% follow-up completion; most felt heard, understood, respected; stimulated ACP beyond ED visit
Pepping et al. (2024) [3]	Netherlands	Mixed methods; *N* = 821 community-dwelling older adults aged ≥ 65 years with acute functional decline	Mean age 83.5 ± 7.7 years; 68% female; 59% multimorbidity; 39% polypharmacy	Transitional care team (transfer nurse ± elderly care physician)	Prevented > 80% unnecessary admissions; revisit rates stable; improved staff awareness and care coordination

Note: ED = Emergency Department; ISAR = Identification of Seniors At Risk

Differences in the organization of healthcare systems were evident across the included studies. For example, studies from the United States, situated within a mixed public-private insurance-based healthcare system, focused on ED-based workflow redesign, communication strategies, and assistive technologies implemented within individual hospitals. In contrast, studies from Ireland and Australia, countries with predominantly publicly funded healthcare, evaluated multidisciplinary team models involving allied health professionals embedded in ED care to support assessment, care coordination, and discharge planning. Studies from the Netherlands reflected that country’s universal health insurance system with strong integration between hospital and community services. These studies emphasized frailty screening and the use of transitional care teams to coordinate care beyond the ED. These contextual differences in healthcare systems, funding structures, staffing models, and geriatric care infrastructure may influence how interventions are implemented and the extent to which findings are transferable across settings. Four main types of interventions were identified and described below.

### Identified interventions

#### Interprofessional programs

Multidisciplinary geriatric care teams in the ED were associated with improved person-centred outcomes and reduced acute care utilization. Pepping et al. [3] studied an ED Transitional Care Team (TCT) that reduced potential admissions among patients with functional decline by nearly 80% without increasing 30-day revisit rates. Patients and caregivers reported clear communication and trust in the TCT’s assessments [3]. A randomized trial of an allied health team (e.g., social work, occupational therapy, physiotherapy) by Cassarino et al. [11] similarly reduced immediate hospital admissions by more than half and shortened ED length of stay (although repeat visits were unaffected). Intervention patients also reported higher satisfaction with their ED care and better functional status at 1- and 6-month follow-ups [11]. Similar benefits were observed in a Geriatric & Palliative ED specialist program (GAP-ED) by Liberman et al. [12] and a Frailty Intervention Team (FIT) by Harper et al. [13], both of which embedded geriatric or frailty specialists into ED workflows. However, challenges remained around sustaining care plans after discharge and how the concept of “frailty” was communicated, particularly the risk that the term can feel discriminatory or stigmatizing. Collectively, these interprofessional programs demonstrated consistent improvements in satisfaction and independence, but highlighted the importance of post-ED coordination to sustain these gains.

#### Redesigning discharge processes

Carayon et al. [14] studied the redesign of ED discharge workflows using human-centred design principles and found the redesigned process feasible and well-received by staff and patients. Interventions included electronic discharge templates, redesigned After Visit Summaries (AVS), teach-back training, and structured follow-up calls. Patients, nurses, and physicians reported improved clarity and perceived safety, particularly valuing more usable AVS printouts. Revisit rates showed little change, but participants reported improved communication. However, workflow barriers such as time pressure at discharge, difficulty completing follow-up calls, and confusion caused by overlapping calls from multiple providers limited the intervention’s impact. These findings highlight that discharge remains a vulnerable transition point requiring dedicated time, improved information design, and better integration with post-ED care providers.

#### Patient comfort

Low-cost, environmental innovations can substantially improve patient experience. Lichen et al. [15] reported that comfort carts stocked with items such as blankets, eye masks, hearing amplifiers, and reading glasses were associated with higher comfort and independence ratings. These were strongly endorsed by staff as facilitating compassionate care. Similarly, Fareed et al. [16] described the routine provision of geriatric assistive devices such as mobility aids (canes, walkers), sensory aids (hearing amplifiers, reading glasses), cognitive engagement tools (large-print puzzles, fidget toys), and personal care items (e.g., bedside commodes) that allowed patients to maintain autonomy and improved their communication with providers, while supporting safer mobility assessments and clinical decision-making. Importantly, these supports were proactively offered to patients to signal attentiveness and preserve dignity. Such interventions required minimal resources but had substantial positive effects on patient satisfaction and workflows.

#### Geriatric screening and assessment

Screening tools and structured conversations targeting geriatric syndromes were generally perceived as acceptable and beneficial. The Acutely Presenting Older Patient (APOP) risk screener (Blomaard et al. [17]) and FIT frailty screening (Harper et al. [13]) were viewed as holistic additions to ED care. However, patients cautioned against stigmatizing use of the label “frailty”. Elder mistreatment screening by Burnett et al. [18] revealed the need for trauma-informed approaches that build rapport before direct questioning and emphasize patient autonomy in follow-up responses. Similarly, a pilot of the ED GOAL intervention, a nurse-led motivational interviewing program for older adults with cognitive impairment and their caregivers by Ouchi et al. [19], was found to be feasible and respectful, with participants feeling heard and supported in articulating care preferences. Although short-term documentation outcomes were limited, these studies suggest that ED-based screening and advance care planning can foster improved communication, safety, and person-centred care when delivered with sensitivity.

### Quality assessment

MMAT tool appraisal of the included 10 studies revealed an overall quality ranging from low to moderate (see Table 2). The single randomized controlled trial by Cassarino et al. [11] used appropriate randomization and demonstrated baseline comparability, but was limited by incomplete outcome data and the inability to blind participants or assessors due to the nature of the intervention. Among the non-randomized studies, Harper et al. [13] relied on a small convenience sample and did not adjust for confounders. Liberman et al. [12] implemented appropriate measures and historical controls but experienced substantial survey attrition. Ouchi et al. [19] was a single-arm pilot with limited representativeness and no adjustment for confounders. The descriptive study by Lichen et al. [15] met most quality criteria, but the risk of non-response bias could not be ruled out. Mixed methods studies had variable quality. Pepping et al. [3] provided a weak rationale for adopting a mixed-methods approach and showed limited integration between quantitative and qualitative findings, while Carayon et al. [14] had weak integration and limited consideration of divergence between methods. Qualitative studies conducted by Blomaard et al. [17], Burnett et al. [18], and Fareed et al. [16] demonstrated high methodological rigour, with coherent designs and findings supported by participant data. Collectively, quantitative studies were constrained by incomplete data and confounding factors, while qualitative and mixed-methods designs offered complementary insights into ED care for older adults.

**Table 2:** Mixed Methods Appraisal Tool for Included Studies

Quantitative randomized controlled study							
Author	Clear questions?	Data address?	Randomization?	Comparable baseline?	Complete data?	Blinded assessors?	Adherence?
Cassarino et al. (2021) [13]	✓	✓	✓	✓	✘	✘	✓
**Quantitative non-randomized**							
**Author**	**Clear questions?**	**Data address?**	**Representative sample?**	**Measurements appropriate?**	**Complete data?**	**Confounders accounted for?**	**Interventions as intended?**
Harper et al. (2023) [16]	✓	✓	✘	✓	✘	✘	✓
Liberman et al. (2020) [18]	✓	✓	✓	✓	✘	✓	✓
Ouchi et al. (2024) [19]	✓	✓	✘	✓	✘	✘	✓
**Quantitative descriptive**							
**Author**	**Clear questions?**	**Data address?**	**Sampling strategy?**	**Representative sample?**	**Measurements appropriate?**	**Low nonresponse bias?**	**Statistical analysis appropriate?**
Lichen et al. (2021) [17]	✓	✓	✓	✓	✓	?	✓
**Mixed methods (MM)**							
**Author**	**Clear questions?**	**Data address?**	**Rationale for MM design?**	**Integration effective?**	**Interpretation adequate?**	**Divergence addressed?**	**Quality criteria met?**
Carayon et al. (2025) [14]	✓	✓	✓	✘	✘	?	?
Pepping et al. (2024) [3]	✓	✓	✘	✓	?	?	✓
**Qualitative**							
**Author**	**Clear questions?**	**Data address?**	**Qualitative approach appropriate?**	**Data collection adequate?**	**Findings supported by data?**	**Interpretation substantiated?**	**Coherence across methods?**
Blomaard et al. (2021) [11]	✓	✓	✓	✓	✓	✓	✓
Burnett et al. (2024) [12]	✓	✓	✓	✓	✓	✓	✓
Fareed et al. (2021) [15]	✓	✓	✓	✓	✓	✓	✓

Note: = Yes; ✘ = No; ? = Can’t tell

## Discussion

### Interpretation of findings

This rapid review found that a range of interventions improved older adults’ perceptions of their ED care experiences. Across diverse settings, interventions that enhanced communication, preserved dignity, and supported shared decision-making were most strongly associated with positive perceptions of care. However, many practices were constrained by workflow pressures, insufficient community linkages, and challenges in sustaining benefits beyond the ED visit. Importantly, low-cost interventions embedded in routine care, such as comfort carts, assistive devices, and communication supports, were highly acceptable to both patients and clinicians.

### Comparison to previous studies

Our findings are consistent with earlier reviews demonstrating that allied health involvement, geriatric consultation, and discharge planning can enhance both experiences and outcomes for older adults in the ED [1, 4]. Berning et al. [4], which focused on patient experience, found that geriatric ED models and care coordination were most commonly associated with improved satisfaction, but highlighted substantial heterogeneity and very low-quality evidence. In contrast, Memedovich et al. [1] examined ED-based interventions targeting utilization (e.g., ED revisits, hospitailization) and reported limited and inconsistent effectiveness, with minimal inclusion of patient perspectives. This rapid review extends prior work by updating the evidence base (2020–2025) to capture innovations developed during and after the COVID-19 pandemic, while further advancing the focus on patient experience established in earlier reviews. Our review also highlighted emerging approaches, including trauma-informed mistreatment screening [18], motivational interviewing-based advance care planning [19], and human-centred discharge redesign [14] – as well as low-cost environmental supports (e.g., comfort carts and assistive devices). These findings suggest that improving older adults’ ED experiences may depend not only on structural geriatric programs but also on integrating geriatric expertise with communication-centred practices and simple environmental supports.

### Strengths and limitations

A key strength of this review is the inclusion of studies conducted during and after the early COVID-19 period, capturing contemporary practices shaped by unprecedented strain on EDs, including heightened public mistrust of the healthcare system, provider burnout, workforce shortages, and temporary ED closures [20–23]. The use of the JBI convergent integrated approach allowed findings from diverse study designs to be synthesized, offering a more comprehensive view of how older adults experienced these interventions.

Nevertheless, several limitations must be acknowledged. Most studies were single-site evaluations with modest sample sizes; the transferability of findings may need to account for differences in funding models, care coordination structures, and cultural expectations. In addition, age thresholds used to define older adults varied across studies (e.g., ≥ 70 years in Blomaard et al. [17]), potentially introducing heterogeneity in the populations studied. However, mean participant ages were generally similar (approximately 80 years), suggesting broadly comparable populations despite differing eligibility thresholds. To support transparency, key demographic details have been added to Table 1. As a rapid review, this study used streamlined methods, including a focused search strategy and a simplified appraisal process (e.g., no manual searching or reference tracing), which may have increased the risk of missing relevant studies. To mitigate this limitation, several health authority project partners were consulted; although they suggested additional articles, none met the predefined eligibility criteria for enhancing patient experience. The exclusion of non-English and grey literature may have further limited the identification of international evidence and ED quality improvement initiatives often published in reports, policy documents, or institutional evaluations.

Methodological limitations were also present in the included studies. Incomplete data, lack of blinding, unmeasured confounding, and short follow-up periods frequently limited quantitative studies. Although qualitative studies were methodologically strong, they were context-specific. These limitations reflect broader challenges in evaluating complex ED interventions and highlight the importance of more rigorous study designs in future research. Cost-effectiveness, caregiver outcomes, long-term functional impacts, and equity-focused analyses were notably underexplored. In addition, few studies used geriatric-specific patient-reported experience measures (PREMs) with established validity evidence, which constrained cross-study comparability.

### Clinical implications

Findings from this review suggest several practical steps that EDs can implement to improve care experiences for older adults.

#### Step 1: Identify geriatric needs early at triage using culturally safe screening approaches

EDs should consider incorporating brief geriatric screening tools (e.g., tools for frailty, functional decline, cognitive impairment, or social vulnerability) during triage or early assessment. Screening for issues such as elder mistreatment or advance care planning should be conducted using culturally safe, trauma-informed approaches that consider patients’ linguistic and cultural needs. Providing multilingual materials and communicating respectfully can improve trust and acceptability among diverse older adult populations. Early identification enables clinicians to prioritize tailored care pathways and engage appropriate interdisciplinary support.

#### Step 2: Engage interprofessional geriatric expertise

Where feasible, EDs should integrate geriatric-focused professionals (e.g., social workers, occupational therapists, physiotherapists, and geriatric or palliative care specialists) into ED workflows. These teams can support functional assessments, discharge planning, and coordination with community services, potentially reducing unnecessary admissions and improving continuity of care.

#### Step 3: Improve communication and shared decision-making

Clinicians should use tailored communication strategies such as simplified after-visit summaries, teach-back techniques, and clear written discharge instructions to ensure patients are not overwhelmed. When possible, caregivers should be involved in discussions, particularly for patients with cognitive impairment or limited health literacy.

#### Step 4: Implement low-cost environmental and comfort supports

Simple interventions such as comfort carts, mobility aids, sensory supports (e.g., hearing amplifiers, reading glasses), and cognitive engagement tools can improve comfort, dignity, and independence while patients wait. These require minimal resources but can substantially enhance the patient experience.

#### Step 5: Strengthen ED-to-community transitions

Before discharge, ED teams should establish clear follow-up plans, coordinate with primary care or community services, and provide accessible discharge instructions. Structured follow-up calls or referrals to transitional care teams may help sustain benefits beyond the ED visit.

### Research and practice implications

Future studies should prioritize multi-site, pragmatic trial designs to assess the effectiveness, implementation, and cost-effectiveness of geriatric ED interventions across diverse health systems. Research should focus on outcomes meaningful to older adults, including independence, delirium prevention, continuity of care, and dignity, and should further develop geriatric-specific PREMs to support their interpretation and routine use. A key strength of this review is its mobilization through a patient-oriented research approach [24]. Older adult patient partners contributed to shaping the initial research questions, reviewing thematic findings through in-person workshops, and participating in meetings with health system leaders and community organizations to guide knowledge translation activities. Through these collaborations, the findings informed priority-setting and ongoing quality-improvement efforts.

## Conclusion

This rapid review shows that a range of targeted, person-centred ED interventions may enhance older adults’ experiences, with some evidence of reduced acute care utilization. Approaches that emphasize person-centred communication, early identification of geriatric needs, and stronger links between ED and community services appear promising. While further research is needed, EDs can already draw on this growing evidence base to implement practical improvements while accounting for local context and resources. Continued efforts grounded in the priorities and lived experiences of older adults will be important to ensure that emergency care remains responsive to the needs of an aging population.

### Appendix:

Search tables for Elderly in the ED rapid review.

**Ovid MEDLINE(R)**.

**Table:** 

#	Query	Results from July 2 2025
1	exp Aged/	3,718,809
2	(aged or elder* or geriatric* or gerontolog* or “old age” or “over 65” or “over sixty-five” or “senior citizen*” or (seniors not “high school”)).tw, kf.	1,212,061
3	((old or older) adj3 (adult* or person* or people or patient or patients)).tw, kf.	439,009
4	1 or 2 or 3	4,589,206
5	Emergency Medicine/ or Emergency Medical Services/ or Emergency Room Visits/ or exp Emergency Service, Hospital/	166,031
6	((emergenc* or “ED” or trauma) adj2 (room* or ward or wards or unit or units or department* or service or treatment* or presentation or visit or visits or setting* or patient or patients or medicine or care or consult* or center* or centre*)).tw, kf.	298,683
7	5 or 6	358,475
8	Patient Satisfaction/ or Patient Preference/	106,784
9	(patient* adj3 (dissatis* or engag* or experienc* or feedback or opinion* or perspective* or prefer* or satisf* or view*)).tw, kf.	387,593
10	8 or 9	445,469
11	4 and 7 and 10 [This is the combination of the Population, Context, and Concept searches]	5,091
12	limit 11 to yr="2020 -Current” [Final results with date limiter applied]	1,724

**CINAHL (EBSCO)**

**Table:** 

#	Query	Results
S12	S4 AND S7 AND S10 [limited 2020-present]	925
S11	S4 AND S7 AND S10	2,892
S10	S8 OR S9	195,268
S9	(MH “Patient Satisfaction”) OR (MH “Patient Preference”)	68,871
S8	XB (patient* N3 (dissatis* or engag* or experienc* or feedback or opinion* or perspective* or prefer* or satisf* or view*))	153,109
S7	S5 OR S6	210,476
S6	(MH “Emergency Service+”) OR (MH “Emergency Medical Services”) OR (MH “Emergency Care+”) OR (MH “Emergency Room Visits”)	129,992
S5	XB ((emergenc* or “ED” or trauma) N2 (room* or ward or wards or unit or units or department* or service or treatment* or presentation or visit or visits or setting* or patient or patients or medicine or care or consult* or center* or centre*))	147,102
S4	S1 OR S2 OR S3	1,273,114
S3	(MH “Aged+”)	996,215
S2	XB ((old or older) N3 (adult* or person* or people or patient or patients))	178,707
S1	XB (aged or elder* or geriatric* or gerontolog* or “old age” or “over 65” or “over sixty-five” or “senior citizen*” or (seniors not “high school”))	420,446

**AgeLine (EBSCO)**.

**Table:** 

#	Query	Results
S12	S4 AND S7 AND S10 [limited 2020-present]	44
S11	S4 AND S7 AND S10	126
S10	S8 OR S9	5,175
S9	DE “Patient attitudes” or DE “PATIENT satisfaction” or DE “PATIENT participation”	1,235
S8	TI (patient* N3 (dissatis* or engag* or experienc* or feedback or opinion* or perspective* or prefer* or satisf* or view*)) OR AB (patient* N3 (dissatis* or engag* or experienc* or feedback or opinion* or perspective* or prefer* or satisf* or view*))	4,261
S7	S5 OR S6	3,415
S6	DE “HOSPITAL emergency services”	805
S5	TI ( (emergenc* or “ED” or trauma) N2 (room* or ward or wards or unit or units or department* or service or treatment* or presentation or visit or visits or setting* or patient or patients or medicine or care or consult* or center* or centre*) ) OR AB ( (emergenc* or “ED” or trauma) N2 (room* or ward or wards or unit or units or department* or service or treatment* or presentation or visit or visits or setting* or patient or patients or medicine or care or consult* or center* or centre*) )	3,340
S4	S1 OR S2 OR S3	133,957
S3	TI ( (old or older) N3 (adult* or person* or people or patient or patients) ) OR AB ( (old or older) N3 (adult* or person* or people or patient or patients) )	66,581
S2	DE “OLDER people” OR DE “CENTENARIANS” OR DE “FRAIL elderly” OR DE “NONAGENARIANS” OR DE “OCTOGENARIANS” OR DE “OLD-old”	32,370
S1	TI ( (aged or elder* or geriatric* or gerontolog* or “old age” or “over 65” or “over sixty-five” or “senior citizen*” or (seniors not “high school”) ) OR AB ( (aged or elder* or geriatric* or gerontolog* or “old age” or “over 65” or “over sixty-five” or “senior citizen*” or (seniors not “high school”) )	90,737

**Web of Science**.

**Table:** 

1	(emergenc* or “ED” or trauma) NEAR/2 (room* or ward or wards or unit or units or department* or service or treatment* or presentation or visit or visits or setting* or patient or patients or medicine or care or consult* or center* or centre*) (Topic)	364,638
2	TS=((patient* NEAR/3 (dissatis* or engag* or experienc* or feedback or opinion* or perspective* or prefer* or satisf* or view*)))	438,948
3	aged or elder* or geriatric* or gerontolog* or “old age” or “over 65” or “over sixty-five” or “senior citizen*” or (seniors not “high school”) (Topic)	5,344,415
4	(old or older) NEAR/3 (adult* or person* or people or patient or patients) (Topic)	603,755
5	#3 OR #4	5,593,348
6	#1 AND #2 AND #5	2,729
7	#1 AND #2 AND #5 [limited 2020-present]	2,480

## Data Availability

Not applicable.
